# Parental Beliefs About the Causes, Treatments, and Medical Assistance for Children With Nocturnal Enuresis in the Eastern Region of the Kingdom of Saudi Arabia

**DOI:** 10.7759/cureus.44557

**Published:** 2023-09-02

**Authors:** Abdullatif K Almaghlouth, Mohammed A Alquraini, Noor A Alsaleh, Mohannad A Almulhim, Thamer K Alhabdan, Mohammad A Alsalman, Abdullah A Alburayh

**Affiliations:** 1 Urology, King Faisal University Hospital, Hofuf, SAU; 2 Pediatrics and Child Health, King Faisal University, Hofuf, SAU

**Keywords:** saudi arabia, perception, parental survey, nocturnal enuresis, behavior, bedwetting

## Abstract

Objective: To assess parental beliefs about the causes, treatment, and necessity for medical assistance for children with nocturnal enuresis (NE).

Method: A self-administered survey questioned parents' beliefs about NE, including causes and at-home behavioral therapy. We evaluated the association between demographic characteristics and the tendency to seek medical advice for NE.

Result: The questionnaire received responses from 1232 individuals, 77.1% of whom were female and 82.9% of whom were 30 years of age or older. Psychological issues (53.5%) and laziness to get up (47.6%) were the most often believed causes of NE. Two frequent at-home behavioral therapies chosen by participants were voiding before bedtime and restricting fluid intake at night (73.4% and 70%, respectively). However, only 6.9% of respondents believe that a bedwetting alarm is an effective treatment. The two most frequently reported reasons for not seeking medical attention were parents' belief that their child will eventually outgrow bedwetting (34.1%) and "parents or children's embarrassment" (21.8%). The chi-squared test was used to evaluate the association between demographic characteristics and seeking medical advice. Participants with a single child were more likely than those with more than three children to take their child to the doctor (61.5% vs. 48.6%, respectively). Also, parents who don't have NE-afflicted children were more open to consider seeking medical advice for NE therapy (if their children developed it in the future) compared to parents who already have children with NE.

Conclusion: Parents in the Eastern region of Saudi Arabia hold various misconceptions about the causes and treatment of NE. Only 52.1% of parents would take their child to see a doctor if wetting the bed and only 48.1% of parents were aware of effective treatments for NE. These results emphasize that healthcare practitioners need to provide proper information to raise public awareness of NE.

## Introduction

Bedwetting, also known as nocturnal enuresis (NE), is a prevalent condition that can affect individuals of various ages and it is characterized by the involuntary release of urine during sleep [1]. There are two types of NE: primary enuresis (urinary incontinence in children who have never achieved dryness) and secondary enuresis (urinary incontinence in children who have been dry for more than six months) [2]. Despite being widely known as a childhood disorder, NE can persist into adolescence and adulthood, making it a significant public health concern [3].

As stated by recent research studies, the prevalence of NE worldwide ranges from 2% to 20%, with higher rates reported among boys than girls [4]. Based on the research studies in nearby countries in the region, it can be stated that the prevalence of NE among children varies across different regions, with reported rates ranging from 11.01% in Iran [5], 17.5%-18.4% in Egypt [6], and approximately 31.2% in Saudi Arabia [7,8]. Furthermore, the studies suggest that the prevalence of NE may be influenced by factors such as age, family history, and exposure to stressful events [8,9].

The causes and risk factors associated with NE are multifactorial but may include genetic predisposition, bladder dysfunction, sleep disorders such as obstructive sleep apnea syndrome (OSAS), psychological issues like anxiety or stress, constipation, hormonal imbalances, diabetes mellitus, and urinary tract infections (UTIs) [3].

The consequences of untreated bedwetting go beyond hygiene concerns and may result in low self-esteem or social isolation due to the shame or stigma attached to this condition. Additionally, it can lead to skin irritation and infection around the genital area as well as increased risks of UTIs if left unmanaged [10].

To address these challenges effectively, we need appropriate management strategies tailored to each patient's unique needs based on their age group and severity level. This may include behavioral interventions such as fluid restriction before bedtime, bedwetting alarms, and medications [3].

Before seeking medical advice, parents frequently implement a variety of simple strategies for dealing with bedwetting, the most common of which are limiting drinks before bed, taking them to the toilet before bedtime, providing positive reinforcement for dry nights, encouraging regular bathroom trips during the day, using protective pants, and showing displeasure [11]. It was noted that parental knowledge about NE is insufficient even though it is a prevalent childhood problem [12]. Many parents believe that bedwetting will go away with age, and as a result, they postpone seeking treatment for bedwetting until it has a significant impact on the child and family [13]. According to a study carried out in Hail City, Saudi Arabia, it was found that the majority of children with enuresis did not receive proper treatment and management [14]. Thus, it is important for parents to seek accurate information about NE and work with their healthcare provider to develop an effective management plan for their child. Therefore, this study aims to investigate Saudi Arabian parents' perceptions of the causes and management of NE. This study will explore how cultural and socioeconomic factors may affect parents' perceptions of the condition and how to treat it, as well as how these perceptions may affect the care their children receive.

## Materials and methods

Population

This cross-sectional study was carried out in the Eastern region of Saudi Arabia, which included six administrative regions (Al-Ahssa, Al-Qatif, Al-Dammam, Al-Khobar, Al-Dhahran, Al-Jubail) to assess the beliefs about the causes, treatments, and necessity for medical assistance for children with NE among parents, and the outcome of interest was to test the hypothesis that parental demographic variables are associated with the decision to take children with NE to a doctor. The data were obtained over the course of one month, in May 2023. The inclusion criteria for the study include parents living in the Eastern region of Saudi Arabia who were able to read and understand Arabic and had children. Parents who did not meet these criteria were excluded from the study.

Survey

The questionnaire used in this study was adapted from Schlomer B et al. [12]. It underwent cross-cultural validation and translation to Arabic. The questionnaire was conducted through an online survey as a total of three pages containing 15 multiple-choice questions. Parental demographics were acquired such as gender, age, nationality, educational level, place of residency, salary income, number of children, and if they had any children with a history of NE. The questionnaire then asked parents to identify the main causes of NE. Also, parents were asked what therapies they would attempt if they had a kid with NE, as well as whether they would take their child to see a doctor. Another question asked parents to explain why they would not bring their child to a doctor. The final set of questions asked participants whether they thought NE should be evaluated by a doctor, if that doctor could assist, and if they knew of any effective therapies for NE.

Statistical analysis 

All statistical analyses were conducted using Statistical Package for the Social Sciences (SPSS) version 25.0 (IBM Corp., Armonk, NY, USA), and a p-value of less than 0.05 was considered statistically significant. Descriptive analysis was done by prescribing frequency distribution and percentage for study variables including parents’ demographical data, beliefs about the causes of NE, beliefs about the treatment of NE, and beliefs about whether a doctor can help with NE. Whether or not a parent selected "take my child to see a doctor" when asked how you would address your child's NE was identified as the outcome of interest in order to test the hypothesis that parental demographics are related to the choice to seek medical care. Cross-tabulation was used to show the association between participant demographics and the decision to seek medical care using Pearson's chi-square test for significance.

## Results

Participant characteristics

A total of 1232 people responded to the questionnaire. The demographics of the survey participants are shown in Table 1. The population was predominantly female, 959 (77.1%), with a high percentage of respondents being 30 years and older, 1021 (82.9). Regarding the educational level, 643 (52.2%) had a bachelor's degree. One hundred and ninety-six participants (15.9%) had a monthly income of less than 4000 SAR, while 461 (37.3%) had a monthly income of more than 12,000 SAR.

**Table 1:** Participants' characteristics

Characteristic	n	%
Gender		
Male	282	22.9
Female	950	77.1
Educational level		
Not educated	0	0
Primary school	11	0.9
Intermediate school	20	1.6
High school	273	22.2
Diploma	157	12.7
Bachelor's	643	52.2
Higher diploma	35	2.8
Master's	66	5.4
PhD	27	2.2
Nationality		
Saudi	1214	98.5
Non-Saudi	18	1.5
Age		
Less than 20 years	25	2
20-29 years	186	15.1
30-39 years	445	36.1
40-49 years	410	33.3
More than 50 years	166	13.5
Family income (monthly)		
Less than 4000 SAR	196	15.9
4000-7999 SAR	280	22.7
8000-11,999 SAR	295	23.9
More than 12,000 SAR	461	37.4
City		
Al-Ahssa	501	40.7
Al-Qatif	343	27.8
Al-Dammam	233	18.9
Al-Jubail	124	10.1
Al-Khobar	20	1.6
Al-Dhahran	11	0.9
Number of children		
1	174	14.1
2-3	507	41.2
More than 3	551	44.7
Having child with nocturnal enuresis		
Yes	558	45.3
No	674	54.7

Descriptive results

Table 2 shows the answers to the following question: What do you think is/are cause(s) of nocturnal enuresis (choose all that apply)? The most commonly reported causes of NE are psychological factors (53.5%), laziness to wake up (47.6%), and NE runs in the family (39.4%). Also, 10.3% of parents reported that they do not know the cause of NE.

**Table 2:** Parental beliefs about causes of nocturnal enuresis

What do you think is/are the cause(s) of nocturnal enuresis?	Number who chose the answer, N = 1232	%
Psychological factors	659	53.5
Laziness to wake up	586	47.6
Nocturnal enuresis runs in the family	485	39.4
Child is a deep sleeper	430	34.9
Child defiant/behavioral problems	277	22.5
Child has a small bladder	272	22.1
Child is seeking attention	233	18.9
I don’t know	127	10.3
Other	26	2.1

Table 3 shows the answers to the following question: If you had a child over the age of 5 who continued wetting the bed, how would you treat him? (choose all that apply)? 73.4% answered “Have my child pee prior to going to sleep”, 70% “Limit my child’s fluid intake before bedtime”, 53.9% “Wake my child up at night and have them go to the restroom”, and 52.1% “Take my child to see a doctor”. On the other hand, bedwetting alarm and punishment of children were uncommon choices at 6.9% and 2.8%, respectively.

**Table 3:** Parental beliefs about treatments of nocturnal enuresis

If you had a child over the age of 5 who continued wetting the bed, how would you treat him?	Number who chose the answer, N = 1232		%
Have my child pee before going to sleep	904	73.4	
Limit my child’s fluid intake before bedtime	863	70	
Wake my child up at night and have them go to the restroom	664	53.9	
Take my child to see a doctor	642	52.1	
Reward my child for dry nights	377	30.6	
Limit my child’s caffeine intake	324	26.3	
No treatment, let my child outgrow it	149	12.1	
Bedwetting alarm	85	6.9	
Reprimand/punish my child for wet nights	34	2.8	
Other	9	0.7	

Table 4 addresses the participant's responses about whether NE requires medical attention, if doctors can be of assistance, and whether they are aware of any treatments. 51.9% of participants were not aware of effective treatments for NE, 87.2% believed that NE can be treated effectively by a doctor, and 86.3% believed that NE should be evaluated by a doctor.

**Table 4:** Parental beliefs about whether a doctor can help with nocturnal enuresis

Question	Number who chose yes, N = 1232	%
Do you believe that bedwetting in children can be treated effectively by a doctor?	1074	87.2
Do you believe that children with bedwetting should be evaluated by a doctor?	1063	86.3
Are you aware that effective treatments are available for children with bedwetting?	593	48.1

Table 5 shows the answers to the following question: If your child suffered from nocturnal enuresis, what would keep you from seeking professional medical care? (choose all that apply)? As for reason(s) that would keep them from seeking professional medical care, the most reported were “Knowing that my child would eventually outgrow bedwetting” (34.1%), followed by “Parent or child embarrassment” (21.8%) and “Fear of invasive tests” (13.7%).

**Table 5:** Reasons that would keep parents from seeking medical care

If your child suffered from nocturnal enuresis, what would keep you from seeking professional medical care?	Number who chose the answer, N = 1232	%
Nothing, I would take my child to the doctor	763	61.9
Knowing that my child would eventually outgrow bedwetting	420	34.1
Parent or child embarrassment	269	21.8
Fear of invasive tests	169	13.7
Nocturnal enuresis is not a significant medical problem	141	11.4
I believe that treatments are ineffective	82	6.7
Expense/costs	60	4.9
Other	8	0.6

Parent demographic variables associated with seeking medical care

Table 6 shows the relation between parents’ demographics and reporting seeking medical advice as a treatment of choice for NE (Taking my child to see a doctor). Considering relations, there was a significant association between the number of children and seeking medical advice as a total of 61.5% of participants having only one child will take their child to see a doctor versus 48.6% of others having more than three children with recorded statistical significance (p = 0.012). Also, parents who do not have NE-afflicted children were more open to considering seeking medical advice for NE therapy (if their children developed it in the future), 60.4%, compared to parents who already have children with NE, 42.1%, with recorded statistical significance (p = 0.000).

**Table 6:** Parents who would treat a child over five years old with nocturnal enuresis by taking the child to a doctor according to participants’ characteristics

Characteristic	I will take my child to see a doctor				p-Value
	Yes		No		
	n	%	n	%	
Gender					0.120
Male	135	47.9	147	52.1	
Female	507	53.4	443	46.6	
Educational level					0.259
Not educated	0	0	0	0	
Primary school	6	54.5	5	45.5	
Intermediate school	11	55	9	45	
High school	127	46.5	146	53.5	
Diploma	93	59.2	64	40.8	
Bachelor's	333	51.8	310	48.2	
Higher diploma	22	62.9	13	37.1	
Master's	34	51.5	32	48.5	
PhD	16	59.3	11	40.7	
Nationality					0.138
Saudi	629	51.8	585	48.2	
Non-Saudi	13	72.2	5	27.8	
Age					0.122
Less than 20 years	11	44	14	56	
20-29 years	101	54.3	85	45.7	
30-39 years	246	55.3	199	44.7	
40-49 years	211	51.5	199	48.5	
More than 50 years	73	44	93	56	
Family income (monthly)					0.999
Less than 4000 SAR	102	52	94	48	
4000-7999 SAR	147	52.5	133	47.5	
8000-11,999 SAR	154	52.2	141	47.8	
More than 12,000 SAR	239	51.8	222	48.2	
City					0.471
Al-Ahssa	252	50.3	249	49.7	
Al-Qatif	187	54.5	156	45.5	
Al-Dammam	121	51.9	112	48.1	
Al-Jubail	66	53.2	58	46.8	
Al-Khobar	8	40	12	60	
Al-Dhahran	8	72.7	3	27.3	
Number of children					0.012
1	107	61.5	67	38.5	
2-3	267	52.7	240	47.3	
More than 3	268	48.6	283	51.4	
Having child with nocturnal enuresis					0.000
Yes	235	42.1	323	57.9	
No	407	60.4	267	39.6	

Seeking medical advice as a treatment of choice for NE was insignificantly higher among females (53.4% vs. 47.9%), residents of Al-Qatif city (54.5% vs. 50.3% for Al-Ahssa), parents with a higher diploma (62.9% vs. 59.3% for PhD), and parents with a monthly family income of 4000-7999 SAR (52.5% vs. 51.8% for others with highest income) with all these demographical characteristics having insignificant statistical association (p > 0.05).

## Discussion

NE, which refers to the involuntary release of urine during sleep in a child who is at least five years old, is a type of incontinence that is well known [15]. It is frequently recognized in school-aged children with severe stressful events, as well as psychological difficulties for children as well as parents [16]. Even though it is well recognized that a variety of conditions lead to this abnormality, many people who suffer from NE keep getting bedwetting after they are older for unknown reasons [17].

The purpose of this study was to gain insight into how parents perceive the causes and management of NE, as well as how cultural and socioeconomic factors may influence parents' perceptions of the condition and how to treat it, along with how these perceptions may influence the care their children receive in the Eastern region of Saudi Arabia. In the current survey, we discovered that the majority of parents thought that psychological factors (53.5%), laziness to wake up (47.6%), and family history (39.4%) were the most frequent causes of NE. According to a study done in Saudi Arabia, the participants thought that the causes of NE were psychological issues (34%), weakness of the muscles in the lower urinary tract (25.3%), hereditary (16.5%), freedom and patience (2.6%), issues with the nerves that control the urinary tracts (5.8%), and causes related to pregnancy and childbirth (0.7%) [18]. Another research by Alhifthy et al. reported that parents believed that the most common causes of enuresis in their study were weakness in the muscles of the lower urinary tract (18.9%), problems or damage to the urinary tract or nerves that control the urinary system (9.1%), psychological problems (8%), UTIs (2.8%), and anemia, hereditary, pregnancy, and birth-related factors [8]. These findings demonstrate how important it is for healthcare professionals to inform parents about the complex nature of NE and the significance of getting appropriate medical care.

Our findings revealed that the majority of participants would follow the following management strategies, having the child pee before going to sleep (73.4%), limiting fluid intake before bedtime (70%), waking the child up at night to use the restroom (53.9%), and taking the child to see a doctor (52.1%). Sherah et al. reported similar findings, showing that parents depended mainly on voiding before sleep (75.3%). Seeking medical advice, on the other hand, was reported by only 38.2% of parents [19]. Child punishment, however, was an uncommon option, accounting for only 2.8% of the parents in our survey. Schlomer et al. found a result that was comparable to ours, reporting that just 2% of parents would punish their child with NE [12]. In contrast, previous research found that around 33.8% of the children were punished for bedwetting [20]. Another research showed that many parents (67.6%) severely punished their enuretic children [21]. It has been noticed that parental characteristics such as being a mother, being a young parent, not understanding child development, having difficulty handling daily stress, or having personality or psychiatric issues are all linked to aggression toward children. Other significant contributing variables are family factors, such as marital issues, a lack of social support, social exclusion, and financial difficulties [22].

Our findings show that enuresis alarms were chosen by 6.9% of patients, and it was comparable to a previous study that stated bedwetting alarms were used by just 6.8% of participants [8]. However, the findings of the current study do not support previous research which showed that enuresis alarms were used for 56.9% of patients [23]. Even though research on enuresis has suggested bed alarms as the basis of treatment along with other behavioral modifications, just a small number of parents in our study would choose it [24,25]. With long-term cure rates of about 50%, it should be noted that alarm therapy is regarded as the first line of treatment for enuresis [26]. It is crucial to keep in mind that some parents might not be aware of more effective options such as bedwetting alarms or drugs. Medical experts should thus educate parents about the variety of practical treatment alternatives.

The significant majority of responders (86.3% and 87.2%, respectively) were in agreement that NE should be assessed and treated by medical professionals. Despite the fact that the majority of parents believed that NE should be evaluated and treated by medical professionals, more than half of the participants (51.9%) were unaware that there are medications that are effective in treating NE. According to a South African study, parents did not seek help and were not informed about monosymptomatic nocturnal enuresis (MNE) treatment choices. Additionally, 42% of parents claimed to be uninformed about MNE treatment options [27]. Another research found that only 32.8% of children with NE had been evaluated by a doctor. Inadequate information or understanding about medical treatments, and cultural differences, might be considered as potential causes for some parent's unwillingness to use medical services despite their availability [28]. Currently, the major method of treating MNE involves bed alarms and desmopressin. Nevertheless, various therapeutic options exist, including tricyclic antidepressants, anticholinergics, and simple non-pharmacological treatments such as water intake regulation [29]. Therefore, parents must be better informed on various NE drugs that might be used for treatment.

The top three reasons for not seeking medical care were the belief that the child would outgrow bedwetting (34.1% of respondents), humiliation felt by either the parents or the child (21.8%), and dread of invasive tests (13.7%). Sarici H, et al. also reported similar findings, stating that some of the parents believed this was a psychosomatic and developmental disease that resolved spontaneously over time and that no therapy was required [30]. Another survey discovered that while 61.3% of parents expressed a likely intention to send their children for therapy, 39% of parents said that they would not seek treatment if the condition did not improve spontaneously [29]. In Korea, Ju et al. observed that parents of children with NE were worried more about the disease's course, and the doctors about interpersonal relations and their patients' low self-esteem. Parents would choose fast-acting, easy-to-use drugs like desmopressin, while doctors would choose long-term solutions [30]. The differences in parental attitudes make it critical to have an accurate understanding of the reasons that can prevent parents from taking their bedwetting children to the doctor. Given these findings, it is important to educate parents about the significance of seeking medical advice to ensure appropriate care for their children's well-being.

While our study provides a valuable understanding of parental beliefs and medical assistance for NE in children of the Eastern region, Saudi Arabia, there are several limitations to consider. First, the study sample included a relative predominance in terms of gender and educational level. The female sample of parents represents 77.1% of participants, and the Bachelor's educational degree of participants accounts for 52.2% which may limit the generalizability of the findings to represent the whole parental population of Eastern Province, Saudi Arabia. Second, the study was conducted only in the Eastern part of Saudi Arabia, so the results may not be representative of attitudes across countries or regions. Third, our study relied on self-reported data from parents, which can be subject to recall bias or social desirability bias. Finally, the study only included parents who volunteered to participate, so the results may be skewed due to selection bias.

## Conclusions

Based on the results of this study, parents in the Eastern region of Saudi Arabia hold various misconceptions about the causes and treatments of NE in children. Only 52.1% of parents would take their child to see a doctor if wetting the bed, and only 48.1% of parents were aware of effective treatments for NE. Psychological factors, laziness to wake up, and a family history of NE were the most commonly reported beliefs that cause NE. The majority of parents reported using behavioral interventions, such as limiting fluid intake before bedtime, having the child pee before going to bed, and waking the child up to use the bathroom, in order to manage NE. For the reasons those parents who did not report they would seek medical care, the majority of them believe that their child will eventually outgrow bedwetting. These findings highlight the need for healthcare professionals to address these misconceptions by providing accurate information to the parents and developing educational materials that are tailored to the cultural beliefs and practices of Saudi Arabian families to promote awareness about evidence-based causes and interventions of NE.

## References

[1] Rincon MG, Leslie SW, Lotfollahzadeh S (2023). Nocturnal Enuresis. StatPearls [Internet].

[2] Bakhtiar K, Pournia Y, Ebrahimzadeh F, Farhadi A, Shafizadeh F, Hosseinabadi R (2014). Prevalence of nocturnal enuresis and its associated factors in primary school and preschool children of khorramabad in 2013. Int J Pediatr.

[3] Vande Walle J, Rittig S, Bauer S, Eggert P, Marschall-Kehrel D, Tekgul S (2012). Practical consensus guidelines for the management of enuresis. Eur J Pediatr.

[4] Yeung CK, Sreedhar B, Sihoe JD, Sit FK, Lau J (2006). Differences in characteristics of nocturnal enuresis between children and adolescents: a critical appraisal from a large epidemiological study. BJU Int.

[5] Makrani AH, Moosazadeh M, Nasehi MM, Abedi G, Afshari M, Farshidi F, Aghaei S (2015). Prevalence of enuresis and its related factors among children in Iran: A systematic review and meta-analysis. Int J Pediatr.

[6] Hamed A, Yousf F, Hussein MM (2017). Prevalence of nocturnal enuresis and related risk factors in school-age children in Egypt: An epidemiological study. World J Urol.

[7] Alsudairy NM, Altamimi AAMT, Alnakhli AA (2021). Nocturnal enuresis in Saudi children: A review. EC Microbiol.

[8] Alhifthy EH, Habib L, Abu Al-Makarem A (2020). Prevalence of nocturnal enuresis among Saudi children population. Cureus.

[9] Alshahrani A, Selim M, Abbas M (2018). Prevalence of nocturnal enuresis among children in Primary Health Care Centers of Family and Community Medicine, PSMMC, Riyadh City, KSA. J Family Med Prim Care.

[10] American Academy of Pediatrics (2023). American Academy of Pediatrics. Bedwetting in Children & Teens: Nocturnal Enuresis. HealthyChildren.org.

[11] Butler RJ, Golding J, Heron J (2005). Nocturnal enuresis: A survey of parental coping strategies at 7 1/2 years. Child Care Health Dev.

[12] Schlomer B, Rodriguez E, Weiss D, Copp H (2013). Parental beliefs about nocturnal enuresis causes, treatments, and the need to seek professional medical care. J Pediatr Urol.

[13] Berry AK (2006). Helping children with nocturnal enuresis: The wait-and-see approach may not be in anyone's best interest. Am J Nurs.

[14] Shahin MM, Zaid Al-Shamary YW, Ahrashed LKM, Al-Motiri SSN, Alhazmi SMA, Alswab WSSA (2017). The epidemiology and factors associated with nocturnal enuresis among school & preschool children in Hail City, Saudi Arabia: A cross-sectional study. Int J Adv Res.

[15] Nevéus T, von Gontard A, Hoebeke P (2006). The standardization of terminology of lower urinary tract function in children and adolescents: Report from the Standardisation Committee of the International Children’s Continence Society. J Urol.

[16] Gür E, Turhan P, Can G (2004). Enuresis: Prevalence, risk factors and urinary pathology among school children in Istanbul, Turkey. Pediatr Int.

[17] Bogaert G, Stein R, Undre S (2020). Practical recommendations of the EAU-ESPU guidelines committee for monosymptomatic enuresis-Bedwetting. Neurourol Urodyn.

[18] Alomani OT, Khalil T (2021). Nocturnal enuresis in primary schools children (6-12 years) of Tabuk city, Saudi Arabia. J Pharm Res Int.

[19] Sherah K, Elsharief M, Barkat N, Jafery A (2019). Prevalence of nocturnal enuresis in school-age children in Saudi Arabia. IJMDC.

[20] Al-Zaben FN, Sehlo MG (2015). Punishment for bedwetting is associated with child depression and reduced quality of life. Child Abuse Negl.

[21] Aliyu OA, Mohammed S, Ocheke IE (2016). Parental perception and response to children with enuresis in JOS. Trop J Nephrol.

[22] Tang CS (2006). Corporal punishment and physical maltreatment against children: A community study on Chinese parents in Hong Kong. Child Abuse Negl.

[23] Al-Zahrani SS (2014). Nocturnal enuresis and its treatment among primary-school children in Taif, KSA. Int J Res Med Sci.

[24] Jain S, Bhatt GC (2016). Advances in the management of primary monosymptomatic nocturnal enuresis in children. Paediatr Int Child Health.

[25] Glazener CM, Evans JH, Peto RE (2005). Alarm interventions for nocturnal enuresis in children. Cochrane Database Syst Rev.

[26] Kuwertz-Bröking E, von Gontard A (2018). Clinical management of nocturnal enuresis. Pediatr Nephrol.

[27] Fockema MW, Candy GP, Kruger D, Haffejee M (2012). Enuresis in South African children: Prevalence, associated factors and parental perception of treatment. BJU Int.

[28] Sarici H, Telli O, Ozgur BC, Demirbas A, Ozgur S, Karagoz MA (2016). Prevalence of nocturnal enuresis and its influence on quality of life in school-aged children. J Pediatr Urol.

[29] Nevéus T, Fonseca E, Franco I (2020). Management and treatment of nocturnal enuresis-an updated standardization document from the International Children's Continence Society. J Pediatr Urol.

[30] Ju HT, Kang JH, Lee SD (2013). Parent and physician perspectives on the treatment of primary nocturnal enuresis in Korea. Korean J Urol.

